# Evaluation of the Young Children with Neurodevelopmental Disability: A Prospective Study at Hamadan University of Medical Sciences Clinics

**Published:** 2013

**Authors:** Afshin FAYYAZI, Leila KHEZRIAN, Zohreh KHERADMAND, Somayeh DAMADI, Ali KHAJEH

**Affiliations:** 1 Pediatric Neurologist, Assistant Professor, Hamaden University of Medical Sciences, Hamadan, Iran; 2 Student of Medicine, Hamadan University of Medical Sciences, Hamadan, Iran; 3 Resident of Otolaryngology, Hamaden University of Medical Sciences, Hamadan, Iran; 4 Assistant Professor of Pediatric Neurology, Zahedan University of Medical Sciences, Zahedan, Iran

**Keywords:** Etiology, Developmental Delay, Speech delay, Children

## Abstract

**Objective:**

Developmental impairment is a common problem in children health that occurs in approximately 5–10% of the childhood population. The aim of this study was to determine the etiologic yield of subspecialists’ evaluation of young children with developmental disability.

**Materials & Methods:**

All children aged between 2 months and 5 years referred over a 15-month period to Hamadan University of Medical Sciences subspecialty services for initial evaluation of a suspected developmental Disability, were enrolled in the present study. Diagnostic yield was determined after the completion of clinical assessments and laboratory tests requested by the evaluating physician.

**Results:**

A total of 198 children (129 boys and 69 girls) were eligible for our study. 108 children had global developmental delay and 90 children had isolated developmental delay. Approximately ¼ of all patients did not have any specific etiology for developmental disability. Cerebral palsy (CP) was the most common clinical syndrome in all patients (41.4%). Hypoxic ischemic encephalopathy (13.8%), brain dysgenesis (13%), genetic disorder (13%), and neurodegenerative diseases (11%) were determined in more than one half of all children with global developmental disability. in our study, “developmental speech delay” was the common cause of isolated speech delay.

**Conclusion:**

Determination of an underlying etiology is an essential part of specialty evaluation of young children with developmental disability. The results of this study were similar closely to the results of other studies.

## Introduction

Developmental impairment is a common problem in children health (1) and occurs in approximately 5–10% of the childhood population (2). As such, it is a frequent reason for referral of a child for specialty evaluation by a pediatric neurologist (1). Based on the percentage of physicians citing neurologic disorders in their practice, developmental delay is one of the top common conditions after ADHD and epilepsy (1). It is demonstrated only about half of the children with developmental impairment are detected before school age (3).

Developmental delays are a group of etiologically heterogeneous, chronic disorders with a documented disturbance in one or more recognized developmental domains; these domains are: motor (gross or fine), speech/language, cognitive, social, and activities of daily living. The disturbance generally must be significant, which means a performance of more than or equal to 2 standard deviations below the mean on a standardized developmental assessment. Global developmental delay (GDD) is described as significant delay in two or more developmental domains (gross motor, fine motor, cognition, speech/language, and personal/social) (2). Its estimated prevalence is 1–3% in children aged less than 5 years (4). Typically, if there is delay in two domains, this can mean that there is a delay across all domains. When a single domain is affected, a gross motor delay or a developmental speech disorder exists (5). Speech delay is a common childhood developmental disorder that affects 3-10% of children (6). The term “delay” should be substituted by “impairment”, because the general population’s perception is that “delay” means something that will get in the end. But the term “impairment” does not suggest that the child will necessarily be normal eventually (7).

Recognition of children at risk for developmental delay and its associated problems can bring about intervention services and family assistance at an early age, that chances for improvement are high (5). Children with developmental disabilities have problems with social skills, memory, and emotional and cognitive functions as well as they have frequent comorbid psychopathology and challenging behaviors (8).

History and examination have the most importance in diagnosis. Studies show that history and examination can establish up to 34% of etiology of developmental delay. Laboratory investigations should be applied after taking a comprehensive history and performing a complete physical examination (9). 

Positive findings were observed in 30-60% of patients with significant developmental delay via newer techniques, such as high resolution computed tomography (CT) and magnetic resonance imaging (MRI) (10). Chromosomal microarray (CMA) is increasingly used for genetic testing of patients with unexplained developmental delay/intellectual impairment (11). The clinical implementation of array comparative genomic hybridization (CGH Array) has been a revolution in the diagnosis of patients with syndromic or nonsyndromic mental retardation and in patients with normal karyotype has detected chromosome abnormalities in up to 17% of cases (12). Inborn errors of metabolism (IEM) has been found to be the causes of developmental delay in a small percentage of these cases (1-5%) (2).

This study aimed to determine common underlying etiologies in our patients and to help us for appropriate management of children with developmental disorders. 

## Materials & Methods

Between January 2011 to March 2012 (a 15-month period), all children aged 2 months to 5 years referred to the Pediatric Neurology Clinics (Imam Khomeini and Besat Hospitals) of the Hamadan University of Medical Sciences, were identified prospectively. 

Eligibility requirements were 2 month to 5 years old of age and referral for the initial evaluation of a suspected developmental disability. Children were excluded from subsequent data analysis if a developmental delay was not confirmed or they could not attend all the necessary diagnostic investigations. The survey included all children with neurodevelopmental disability (according to initially abnormal Denver II test) and finally confirmed by clinical judgment of a child neurologist (13). Besat and Emam Khomeini centers are the main referral centers for pediatric neurologic disorders in the west of Iran with near 200 and 600 attending patients monthly. This study was approved by the Research Committee of Hamadan University of Medical Sciences.

In this study we considered the term “Developmental Disability” for all patients with developmental delay or regression. Cerebral palsy (CP) was considered as a group of permanent disorders of movement and posture, causing activity limitation and static encephalopathy in the developing fetal or infant brain. Intrapartum asphyxia was diagnosed on the basis of a combination of a history and objective documentation of different intrapartum complications. Autism was considered to be present when repetitive behaviors, desire for sameness, avoidance of eye contact, social isolation, and lack of imaginative play were characteristics that were evident on either history or neurodevelopmental examination. Chromosomal etiology defined as a genetic abnormality that is obvious on karyotype analysis. Genetic etiology indicates all other syndromes with known genetic cause. Cerebral dysgenesis was defined with features on neuroimaging. All children that were late talker and were normal in other developmental aspects, with familial history of speech delay, were considered as “Developmental Speech Delay”. All children that had motor delay and were normal in other aspects and physical examination, with familial history of motor delay and normal paraclinical study, were considered as “Familial Motor delay” At initial assessment, an information sheet was completed on each subject for all patients by a child neurologist. Specific laboratory testings were performed on a case-by-case basis (14) and brain imaging, metabolic screening, EEG, EMG– NCV, ABR(16), audiometry, VEP, and genetic tests 

**Table 1:** The Etiologies of Neurodevelopmental Disability in Studied Groups

	**Global Delay**	**Isolated Motor Delay**	**Isolated Speech Delay**
Hypoxic Ischemic Encephalopathy	15	23	1
Kernicterus	3	6	-
Viral Encephalitis	2	-	-
Head Trauma	2	1	-
Metabolic Disorders	7	1	-
Neurodegenerative Disorders	12	-	-
Brain dysgenesis	14	3	-
Genetic disorders	14	-	1
Chronic systemic disorders(cystic fibrosis)	1	-	-
Congenital brain mass	1	-	-
TORCH (Toxoplasmosis)	1	-	-
Pervasive developmental Disorders	2	-	2
Congenital Muscular Dystrophy	1	-	-
Spinal Muscular Atrophy	-	1	-
Muscular dystrophy	-	1	-
Congenital Myopathy	-	1	-
Landau Kleffner syndrome	-	-	3
Developmental speech Delay	-	-	10
Familial Motor Delay	-	6	-
Hyper mobility Syndrome	-	2	-
Neurocutaneous syndromes(Tuberous Sclerosis)	2	-	-
meningitis	1	-	-
Neonatal Hydrocephaly	-	-	1
Epileptic Encephalopathy	1	-	-
Unknown	29	12	13

(karyotyping and single gene study such as MECP2), were selected according to primary clinical diagnosis. We did not evaluate Intellectual Quotient (IQ) in our patients. At least 6-month follow-up was done to confirm diagnosis.

## Results

A total of 198 children (129 boys and 69 girls) with the mean age of 26.18±16.29 months (a range age of 2 months to 65 months) were eligible for our study. The chief complaints and histories of patients were in 87% developmental delay and in 13% developmental regression.

108 children (54.5%) had global developmental disability (80.5% delay and 19.5% regression) and 90 children (45.5%) had isolated developmental disability (94.5% delay and 5.5% regression). 

Patients with isolated developmental disability had 29.7% motor, 15.6% speech and 1% cognition problems.

The parents of children were relative in 38% of children and the history of developmental disability in family was positive in 30% of patients. 31.3% of children had the history of seizure during their life.

CP was the most common clinical syndrome in all patients (41.4%). CP was seen in 42.6% of cases in

global developmental disability group and 63.1% of cases in isolated motor developmental disability group. Developmental speech delay was the most common clinical syndrome in isolated speech disability group (38.7%).

Hypoxic ischemic encephalopathy (HIE) was the cause of developmental delay in 22.6% of all children (13.8 % of global and 41 % of motor delay) (Table1). 

Metabolic disorder was demonstrated in 8 patients (4%), which included 3 Mucopolysaccharidoses, 2 maple syrup urine diseases, 2 Phenylketonuria, 1 galactosemia and 1 renal tubular acidosis. 12 children had clinical manifestations of neurodegenerative disorders, but definite diagnosis 

was determined in 4 patients (1 Adrenoleukodystrophy, 1 neuronal ceroid lipofuscinoses, 1 Krabbe disease, and 1 infantile bilateral striatal necrosis). In patients with autistic feature (17 children) etiologies were determined in 35.2% (HIE, brain dysgenesis, metabolic disorders) and in the others, etiologies were unknown. 

9 children had specific genetic disorders (2 Down syndrome, 1 trisomy 18, 1 Rubinstein-Taybi syndrome, 1 Cockayne syndrome, 1 Rett syndrome, 1 tuberous sclerosis, 1 Neurofibromatosis, 1 Spinal Muscular Atrophy disorder).

In 25.7% of all patients, no specific etiology was identified for developmental disability (25.9% in global, 21% in motor and 35.5% in speech).

## Discussion

In this prospective descriptive study, an etiology was determined in about ¾ of children with global

developmental disability but in patient with global developmental disability and autistic feature, this rate was about one third. Hypoxic ischemic encephalopathy (13.8%), brain dysgenesis (13%), genetic disorder (13%) and neurodegenerative diseases (11%) were specified etiologies in one half of all children with GDD. 

A subspecialists’ evaluation of young children with GDD in Canada demonstrated the presence of co-existing autistic traits was related with significantly decreased diagnostic yield. In that study, four diagnoses (cerebral dysgenesis, hypoxic-ischemic encephalopathy, toxin exposure, chromosomal abnormalities) were responsible for 77% of the diagnoses made (14). Since in the present study we did not assay serum lead level in patients with GDD therefore toxin exposure was not a demonstrated cause in our survey. 

In a similar cohort study in India, the etiological yield in young children with GDD was 73% and four diagnostic categories including Down syndrome (20%), HIE (15%), multiple malformation syndromes (14%), and cerebral dysgenesis (11%) were the most common causes of GDD (15). The result of this study was very similar to our finding but Down syndrome was not a common reason for referral in our patients. Inborn error of metabolism was not a common reason for GDD in our study, but in situations where developmental delay is associated with specific finding such as hypotonia, regression, eye abnormalities or hepatosplenomegaly, investigations are valuable (2).

CP was the most common clinical presentation in our patients (41.4%). we considered all children with static encephalopathy and motor impairment as CP. In a retrospective study conducted at a tertiary care centre in north India, CP was demonstrated in 12.7% of patients but authors considered non chromosomal syndromes (9.5%), central nervous system structural defects (7.4%), and environmental insults (2%) in different categories (17).

Speech development delay may be a symptom of other diseases, such as mental retardation, expressive language disorder, hearing loss, autism, and CP. Speech delay may be secondary to maturation (developmental language delay). Maturation delay is responsible for a significant percentage of late talkers. Family history of “late bloomers” is often present in this condition (6). Despite mental retardation is the most common cause of the chief complaint of “speech delay”, accounting for more than 50% of cases (6), in our study “developmental speech delay” was the common cause of isolated speech delay. In our method, all children with both cognition and speech delay were considered to be GDD. 

**In conclusion, **Determination of an underlying etiology is an essential part of specialty evaluation of young children with developmental disability and helps for appropriate rehabilitation, family counseling and management of any associated medical conditions. 
